# Acute External Iliac Artery Occlusion After Posterior Total Hip Arthroplasty: A Case Report

**DOI:** 10.1155/cro/7440156

**Published:** 2026-10-05

**Authors:** Austin Post, Jessica Nelson, Michael Miller

**Affiliations:** ^1^ Department of Orthopedic Surgery The University of Arizona Tucson Arizona USA arizona.edu

**Keywords:** acute limb ischemia, case report, external iliac artery, total hip arthroplasty

## Abstract

Acute limb ischemia is a rare but potentially limb‐threatening complication following total hip arthroplasty. Although vascular injuries have been described in the literature, proximal arterial occlusion at the level of the external iliac artery after a primary posterior total hip arthroplasty, to our knowledge, has not been previously reported. We present the case of a 74‐year‐old woman who developed acute limb ischemia approximately 3 h after undergoing an primary posterior total hip arthroplasty without any intraoperative complications. On postoperative examination, she was noted to have diminished distal perfusion of the operative extremity. Upon return to the operating room, angiography revealed an acute occlusion of the external iliac artery. The patient underwent emergent thromboembolectomy, which successfully restored arterial flow. No underlying vascular disease or hypercoagulable condition was identified on further evaluation, and a positional or compression‐related mechanism was hypothesized as the most likely mechanism. This case highlights an underrecognized but serious complication that may occur even in routine total hip arthroplasty. It underscores the importance of meticulous intraoperative positioning, heightened awareness of potential vascular compromise, and routine postoperative neurovascular assessment. Early recognition of ischemic signs and prompt surgical intervention are critical to preventing irreversible limb damage and optimizing patient outcomes.

## 1. Introduction

Acute limb ischemia (ALI) is a rare and limb‐threatening complication after total hip arthroplasty (THA). The rate of vascular complications following THA is reported between 0.2% and 0.3% [1, 2]. Causes of ALI include vascular injury, traction, retractor or positioner compression, thrombosis, anatomic variation, and atherosclerosis [3–6]. Postoperative signs, including pulselessness, diminished pulses, persistent pain, pallor, paresthesia, or paralysis, require immediate recognition, as prompt diagnosis and intervention are essential to prevent irreversible ischemia or limb loss.

As outpatient THA increases in an aging population, awareness of this complication is critical. Although external iliac artery occlusion has been described after complex revision THA and, in one instance, after an anterior approach, to our knowledge it has not been reported following a primary posterior THA without intraoperative difficulty or vascular injury recognized at the time of surgery [7–9]. Although the precise etiology is unclear, a positional or compression‐related mechanism is hypothesized. Thrombosis extended distally to the common femoral, profunda, superficial femoral, popliteal, and anterior tibial arteries. This case was written in accordance with the CARE (CAse REport) guidelines to ensure transparent and complete reporting; the complete CARE checklist is provided as a supporting information.

## 2. Case Presentation

A 74‐year‐old woman with a medical history only notable for hyperlipidemia (total cholesterol 230 mg/dL, LDL 148 mg/dL) presented with severe end stage right hip osteoarthritis. She weighed 54 kg and underwent elective right THA under general anesthesia. Preoperative radiographs (Figure 1) showed complete loss of the joint space with severe subchondral sclerosis and subchondral cysts most noticeable in the superior acetabulum.

**Figure 1:**
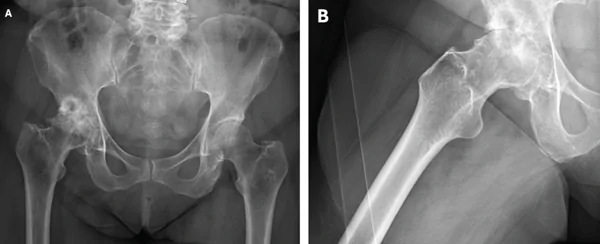
(A,B) Anteroposterior pelvis and lateral radiographs of the right hip demonstrating advanced osteoarthritis with joint space narrowing and cystic changes within the superior acetabulum.

The patient was positioned in the lateral decubitus position using Stulberg positioners, and a standard posterior approach was performed. The superior acetabular cyst was curetted and backfilled with morselized femoral head autograft, which was reverse reamed into the defect. A press‐fit acetabular shell was placed with supplemental screw fixation and a high‐wall liner. A standard‐offset collared femoral stem was placed. Immediate postoperative radiographs are shown in Figure 2.

**Figure 2:**
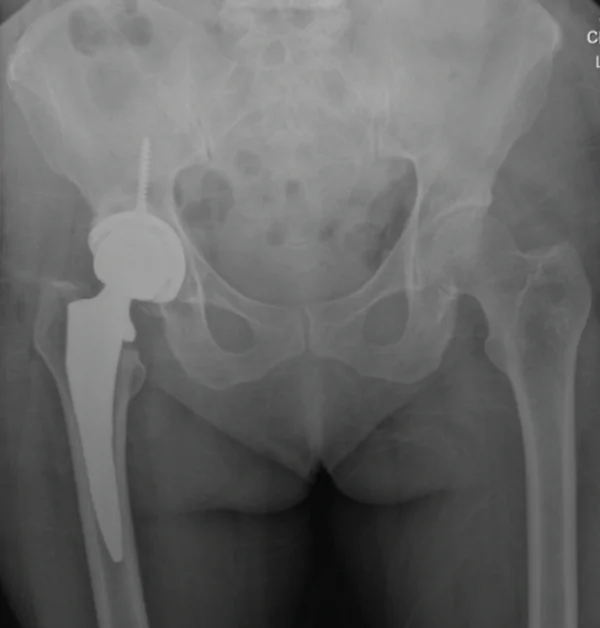
Immediate postoperative radiograph demonstrating an uncemented total hip arthroplasty with a single acetabular screw in place.

Approximately 3 hours postoperatively, the patient developed severe leg pain, decreased dorsiflexion, and absent Doppler signals in the dorsalis pedis and posterior tibial arteries. The limb was cool with dorsal foot mottling, and no thigh hematoma was appreciated. Vascular surgery was urgently consulted, who diagnosed her with Stage 2b ALI based on lack of arterial Doppler signal, forefoot sensory changes, and mild motor impairment. The patient proceeded directly to intraoperative angiography followed by emergent open thromboembolectomy via a groin incision. Per the vascular surgery team, an acute appearing thrombus was found that spanned from the external iliac to proximal anterior tibial artery. They noted normal‐appearing intima without injury or significant atherosclerotic disease. Flow was restored to the external iliac, common femoral, profunda femoris, superficial femoral, popliteal, and anterior tibial arteries. Intraoperative angiography demonstrated external iliac artery occlusion with distal propagation (Figure 3A,B, red star) and restoration of flow after thrombectomy (Figure 3C,D). No underlying peripheral arterial disease was identified.

**Figure 3:**
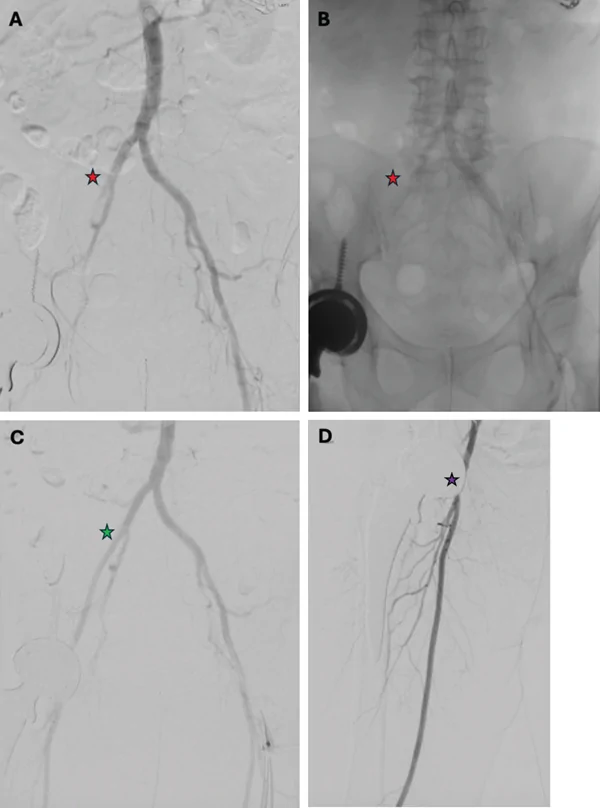
(A–D) Angiographic images demonstrating occlusion of the external iliac artery (A and B, red star), followed by restoration of flow in two projections. Panel (C) shows flow restoration with contrast distal to the previous site of occlusion (green star). Panel (D) demonstrates re‐established perfusion with contrast flow past the implant into the femoral artery, albeit with a small segment that appears to lack contrast at the level of the acetabulum due to implant overlap (purple star).

Postoperatively, she received intravenous therapeutic heparin for 48 h, then transitioned to apixaban upon discharge for 3 months. Inpatient studies including hematologic labs (PT, INR, and fibrinogen), echocardiogram, and electrocardiogram were unremarkable. Outpatient vascular and hematology evaluations were normal, including a negative coagulopathy and antiphospholipid antibody workup. At 7 months postoperative, an AP pelvis radiograph demonstrated a stable implant without evidence of subsidence, and the patient was clinically doing well without complaints.

### 2.1. Timeline

• Day 0: Elective THA via posterior approach performed under general anesthesia with Stulberg positioners; acetabular cyst grafted, press‐fit shell with single screw, and standard offset collared femoral stem.
• 3 hours postoperative: Onset of severe leg pain, decreased dorsiflexion, and absent Doppler signals (dorsalis pedis, posterior tibial) with mottled skin; vascular surgery consulted urgently and diagnosed with Stage 2b ALI.
• 4 hours postoperative: Taken directly to hybrid operating room for diagnostic angiography, which demonstrated occlusion starting at external iliac artery.
• 5 hours postoperative: Conversion to open thromboembolectomy performed with flow restored from external iliac and distal; confirmed with completion angiography.
• Postoperative days 0–2: Admitted postoperatively and received intravenous therapeutic heparin, hematologic labs, echocardiogram, and electrocardiogram, all returning unremarkable.
• Postoperative day 3: Discharged home on apixaban for 3 months.
• Outpatient follow up: Vascular surgery and hematology evaluations normal, including negative coagulopathy and antiphospholipid antibody workup. Postoperative vascular ultrasounds demonstrate normal arterial flow of the affected limb.
• 7 month follow up: Orthopedic surgery evaluation with the patient clinically doing well without further complication, ambulating unassisted; radiographs demonstrated stable implant without subsidence.

## 3. Discussion

ALI following primary THA is exceedingly rare but potentially catastrophic, with risk of limb loss and mortality [1–4, 7–11] . Although uncommon, it remains a serious and potentially catastrophic complication that may result in limb loss, amputation, and mortality. To our knowledge, this represents the most proximal arterial occlusion reported after a routine primary THA for osteoarthritis performed through a standard posterior approach. A compression‐related mechanism caused by anterior and medial pressure from the Stulberg positioning pad on the external iliac artery is one potential etiology.

External iliac artery thrombosis has been described once in a complex primary THA performed through an anterolateral approach in a patient with longstanding femoral neck fracture and significant limb‐length discrepancy requiring extensive soft‐tissue release; occlusion was identified 6 h postoperatively and attributed to aberrant anatomy and limited anterior acetabular visualization [11]. Additionally, a single report described external iliac artery injury during a direct anterior approach in a medically complex patient with an underlying iliac aneurysm [7].

The precise etiology of ALI in this patient remains uncertain; however, a positioning‐related mechanism is suspected, and to our knowledge, has not been described in uncomplicated primary posterior THA in the literature. A plausible explanation is excessive anterior and medial compression of the femoral neurovascular structures by the Stulberg positioning device. The proper positioning of the anterior Stulberg is at the level of the anterior superior iliac spine (ASIS). If positioned too medial and inferior as demonstrated in Figure 4, sustained pressure could reduce flow through the external iliac artery, particularly with the limb maintained in flexion, adduction, and internal rotation. This patient′s low body weight (54 kg) may be mechanistically relevant. Thin patients have less subcutaneous and soft‐tissue padding overlying the anterior pelvis, potentially allowing positioner pressure to be transmitted more directly onto the underlying neurovascular structures. In Figure 4, it is clear that when the anterior positioner is more medial and inferior to the ASIS, the pressure is right at the level of the external iliac artery, where the occlusion was found in this patient. Whether patients with centripetal obesity face a similarly increased risk is less clear. Redundant abdominal and thigh soft tissue could necessitate deeper and more medial placement to maintain positioner stability, which may theoretically offset any protective effect of additional soft‐tissue padding. This relationship has not been established and warrants further investigation.

**Figure 4:**
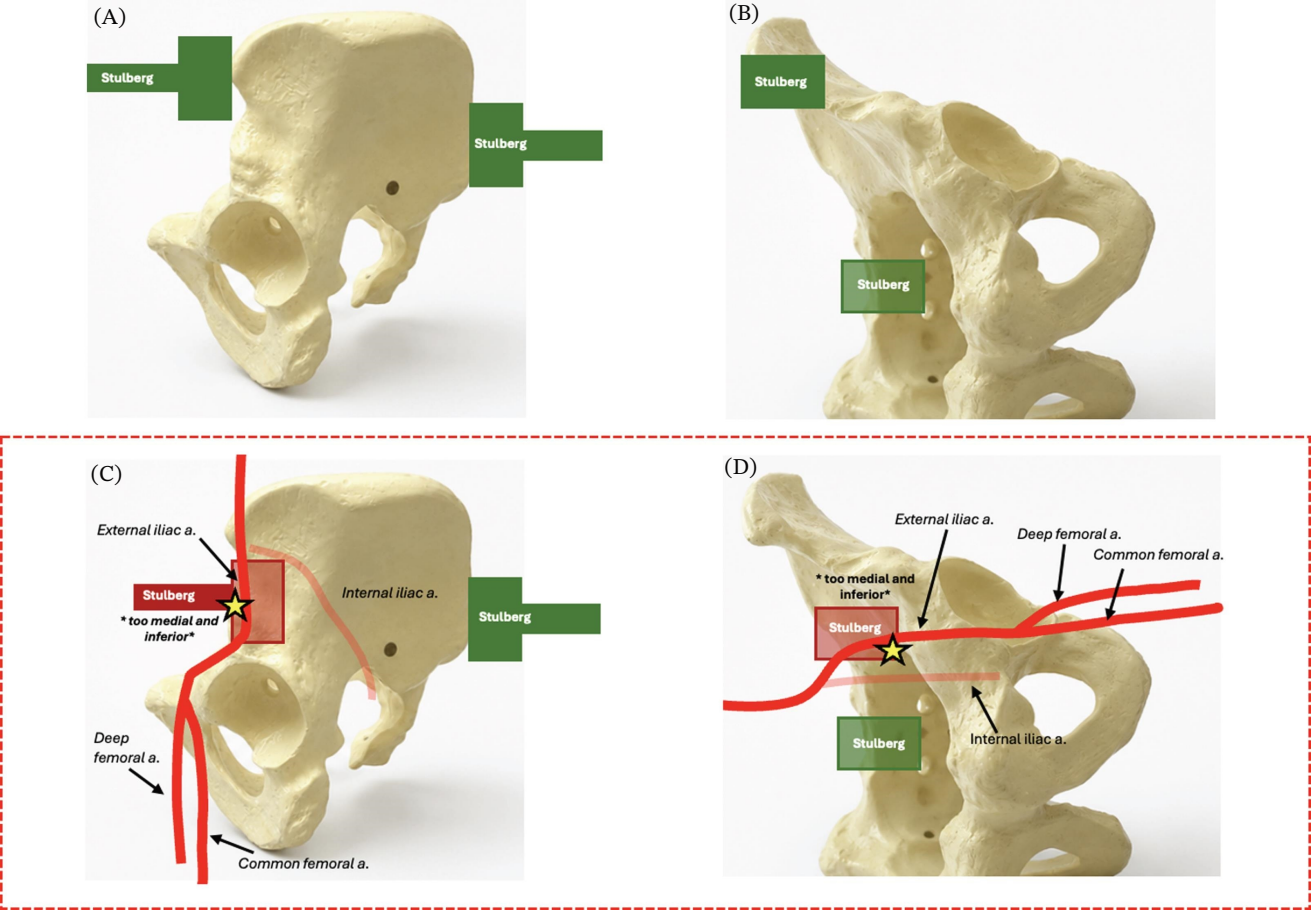
Positioning of the Stulberg hip positioners for lateral decubitus posterior total hip arthroplasty. Correct positioning is shown in (A) the sagittal and (B) coronal planes, with the anterior positioner at or just proximal to the anterior superior iliac spine (ASIS) and the posterior positioner at the level of the upper sacrum. A possible positioning error is shown in panels (C) and (D) (outlined in red): when the anterior positioner is placed too medially and inferiorly, the compression vector is directed onto the external iliac artery, risking vascular compromise.

Outpatient vascular and hematologic evaluations were unremarkable. Although the patient had hypercholesterolemia (HDL 62 mg/dL, LDL 148 mg/dL), angiography demonstrated no arterial calcification or significant occlusive disease. Given the proximal location of the occlusion, retractor‐related injury is unlikely, further supporting a compression‐related etiology. To our knowledge, an association between Stulberg positioning devices and vascular complications during THA has not been previously reported.

In addition to retractor placement and patient positioning, inadvertent vascular injury during acetabular screw placement must be considered. An excessively anterior or superior screw trajectory, anatomic variation, or unrecognized acetabular deformity can result in external iliac artery injury. However, this mechanism is unlikely in the present case, as such injuries typically manifest intraoperatively or immediately postoperatively with hypotension, groin swelling, or hemorrhagic instability. Although theoretical intimal injury from screw proximity could result in delayed thrombosis without frank perforation, no evidence of arterial penetration or direct injury was identified at the time of surgical revascularization.

Femoral vessel injury may also occur during femoral canal preparation when the hip is placed in extreme flexion, adduction, and internal rotation. This positioning can tension the femoral artery or compress it against the femoral head or acetabular rim, particularly in thin patients or those with advanced atherosclerosis. Intimal disruption from traction or limb manipulation has been described as a potential mechanism of injury. However, this explanation is less likely in the present case. The occlusion was identified proximal to the common femoral artery at the level of the external iliac artery, and angiography did not demonstrate preexisting atherosclerotic plaque or underlying vessel disease. These findings suggest an alternative mechanism rather than plaque destabilization or distal traction‐related intimal injury. Careful review of preoperative imaging for calcified or tortuous vessels is important. Taken together, the intraoperative finding of normal‐appearing intima without atherosclerotic disease, the absence of any recognized retractor or screw‐related injury, and the proximal location of the occlusion at the external iliac artery make these alternative mechanisms unlikely. We suggest the circumstances are most consistent with an extrinsic compression injury from the positioning device, though this remains a hypothesis rather than a confirmed mechanism.

Similarly, severe acetabular deformity—whether from prior trauma, dysplasia, or inflammatory arthropathies such as rheumatoid arthritis—may distort local anatomy and increase the risk of vascular compromise during THA [4, 5].

A key takeaway from this case—particularly in an era of increasing outpatient THA performed under spinal anesthesia—is the importance of thorough patient and caregiver education. Because routine postoperative vascular checks may not occur in ambulatory settings, and spinal or epidural anesthesia may mask early sensory changes, patients and families should be counseled before discharge to monitor for severe pain, pallor, coolness, numbness, or motor weakness and to seek immediate care if these occur. Postoperative dressings such as compression stockings or pneumatic devices can further obscure visual inspection of the limb, reinforcing the need for explicit counseling.

## 4. Clinical Message

Arthroplasty surgeons can reduce the risk of catastrophic vascular complications by emphasizing vigilance before, during, and after THA. Preoperative, patients should be counseled on red flag symptoms—such as severe pain, pallor, coolness, numbness, or motor changes—and instructed to seek immediate care if they occur. Intraoperatively, meticulous attention to positioning and padding is essential, specifically in patients with low body weights. Providers should remain vigilant that positioning devices or limb orientation may contribute to occult vascular compression. Postoperative, nursing staff should be educated to perform serial vascular examinations, particularly in the early recovery period or outpatient setting.

## Funding

No funding was received for this manuscript.

## Consent

The patient was informed that data concerning the case would be submitted for publication and the patient agreed.

## Conflicts of Interest

The authors declare no conflicts of interest.

## Supporting information


**Supporting Information** Additional supporting information can be found online in the Supporting Information section. CARE checklist of information to include when writing a case report.

## Data Availability

The data that support the findings of this study are available from the corresponding author upon reasonable request.
