# Supplementary material for: Acute External Iliac Artery Occlusion After Posterior Total Hip Arthroplasty: A Case Report
Source: Case Rep Orthop. 2026 Oct 5;2026:7440156. doi: 10.1155/cro/7440156 (PMC13636141; doi:10.1155/cro/7440156)
Supplement: Supplementary file 1 [file CRO-2026-7440156-s001.docx]

# CARE Checklist

*Manuscript: Acute External Iliac Artery Occlusion After Posterior Total Hip Arthroplasty: A Case Report*

| **Topic** | **Item No.** | **CARE Checklist Item** | **Location in Manuscript** | **Reported (Y/N/NA)** |
| --- | --- | --- | --- | --- |
| Title | 1 | The area of focus and 'case report' should be in the title. | Title | Yes |
| Key words | 2 | 2 to 5 key words that identify diagnoses/interventions in this case report. | Below abstract | Yes |
| Abstract | 3a-e | Introduction, case, conclusion, plus what is new. | Abstract | Yes |
| Introduction | 4 | Brief background summary referencing scientific literature. | Introduction | Yes |
| Patient Information | 5a-d | De-identified demographics, main concerns/symptoms, medical/family/psychosocial history, relevant genetic information. | Case Presentation | Yes |
| Clinical Findings | 6 | Relevant physical exam and clinical findings. | Case Presentation | Yes |
| Timeline | 7 | Dates and times of relevant past/current interventions organized in a table or figure. | Patient Timeline | Yes |
| Diagnostic Assessment | 8a-d | Diagnostic methods, challenges, reasoning, and prognostic characteristics where applicable. | Case Presentation | Yes |
| Therapeutic Intervention | 9a-c | Types, administration, and changes of intervention with rationale. | Case Presentation | Yes |
| Follow-up and Outcomes | 10a-d | Clinician/patient-assessed outcomes, important follow-up test results, adherence, adverse events. | Case Presentation | Yes |
| Discussion | 11a-d | Strengths/limitations of management, relevant literature, rationale for conclusions, primary lessons. | Discussion | Yes |
| Patient Perspective | 12 | Patient's perspective on treatment, where appropriate. | N/A - not obtained for this report | N/A |
| Informed Consent | 13 | Statement of informed consent; patient consent for photographs if applicable. | Statement of Informed Consent | Yes |
